# Characterization and Subcellular Targeting of GCaMP-Type Genetically-Encoded Calcium Indicators

**DOI:** 10.1371/journal.pone.0001796

**Published:** 2008-03-19

**Authors:** Tianyi Mao, Daniel H. O'Connor, Volker Scheuss, Junichi Nakai, Karel Svoboda

**Affiliations:** 1 Cold Spring Harbor Laboratory, Cold Spring Harbor, New York, United States of America; 2 Howard Hughes Medical Institute, Janelia Farm Research Campus, Ashburn, Virginia, United States of America; 3 Laboratory for Memory and Learning, RIKEN Brain Science Institute, Wako-shi, Saitama, Japan; University of Southern California, United States of America

## Abstract

Genetically-encoded calcium indicators (GECIs) hold the promise of monitoring [Ca^2+^] in selected populations of neurons and in specific cellular compartments. Relating GECI fluorescence to neuronal activity requires quantitative characterization. We have characterized a promising new genetically-encoded calcium indicator—GCaMP2—in mammalian pyramidal neurons. Fluorescence changes in response to single action potentials (17±10% ΔF/F [mean±SD]) could be detected in some, but not all, neurons. Trains of high-frequency action potentials yielded robust responses (302±50% for trains of 40 action potentials at 83 Hz). Responses were similar in acute brain slices from *in utero* electroporated mice, indicating that long-term expression did not interfere with GCaMP2 function. Membrane-targeted versions of GCaMP2 did not yield larger signals than their non-targeted counterparts. We further targeted GCaMP2 to dendritic spines to monitor Ca^2+^ accumulations evoked by activation of synaptic NMDA receptors. We observed robust ΔF/F responses (range: 37%–264%) to single spine uncaging stimuli that were correlated with NMDA receptor currents measured through a somatic patch pipette. One major drawback of GCaMP2 was its low baseline fluorescence. Our results show that GCaMP2 is improved from the previous versions of GCaMP and may be suited to detect bursts of high-frequency action potentials and synaptic currents *in vivo*.

## Introduction

Understanding the function of neural networks will require the ability to monitor action potentials and synaptic activity in populations of identified neurons. In mammalian pyramidal neurons, action potentials trigger a transient calcium influx though voltage-gated calcium channels that can occur both at the soma and in the dendrites following backpropagation of the action potential [1]–[4]. Action potential (AP)-evoked calcium transients have been used extensively to measure neuronal spiking activity *in vitro* and *in vivo* [5]–[9]. In addition, NMDA receptor-dependent calcium accumulation in dendritic spines has been used to monitor the activity of individual synapses [10]–[16].

The vast majority of calcium imaging experiments have employed synthetic calcium indicators, which permit measurements of AP- and synaptically-evoked calcium transients. However, genetically-encoded calcium indicators (GECIs) provide advantages over synthetic indicators [17]. They allow: (1) monitoring activity among genetically-defined subsets of cells, (2) measuring calcium dynamics in specific subcellular compartments, and (3) long-term calcium imaging in vivo.

GECIs are engineered based on either changes in the florescence intensity of a single fluorophore, or changes in fluorescence resonance energy transfer (FRET) efficiency. For example, the GCaMP family of GECIs is composed of a single circularly permuted GFP with calmodulin (CaM) and its binding peptide myosin light-chain kinase M13 linked to its C- and N-termini, respectively. Upon calcium binding, conformational changes in the CaM/M13 complex cause a fluorescence change in the circularly permuted GFP-based fluorophore [18]. FRET-based GECIs are based on two designs. In the cameleon family [19], a calcium-dependent increase in FRET between a CFP and YFP FRET pair is coupled by the binding of calmodulin to the M13 peptide. The troponin family of sensors utilizes the skeletal muscle calcium sensor troponin C (TnC). Binding of calcium to troponin causes a conformational change that increases FRET between CFP and YFP [20]. Since endogenous TnC, unlike calmodulin, is not expressed in neurons, TnC-based sensors may show reduced interference with endogenous signal transduction processes in neurons [21]. Recently developed GECIs have provided improved brightness, dynamic range, speed, pH- and Mg^2+^- sensitivity, thermal stability and folding efficiency [17], [21]–[25]. Several lines of mammalian GECI transgenic animals have been engineered [23]–[26], but the small signal levels in these mice [25]–[27] have so far not permitted widespread use for in vivo physiology. Better results have been achieved in invertebrate systems [28]–[30].

**Figure 1:**
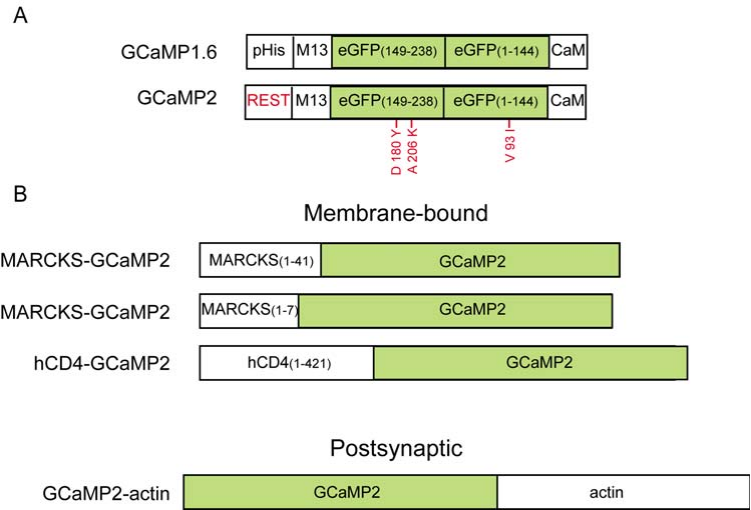
Domain structures of the GCaMP-family of genetically encoded calcium indicators (GECIs) and fusion constructs. A, Domain comparisons of GCaMP2 and GCaMP1.6; red labels indicate the differences. B, Constructs for subcellular targeting of the GECIs.

To understand the advantages and limitations of each GECI for measuring neuronal activity a quantitative comparison of GECI signals under identical experimental conditions is required. In pilot studies we screened through several members of the latest generation of GECIs and identified GCaMP2 [24], the latest member of the GCaMP family, as particularly promising. We evaluated several versions of GCaMP2 (Figure 1), focusing on its suitability for monitoring action potentials and NMDA-R activation in single spines in mammalian pyramidal neurons. We found that GCaMP2, compared to its predecessors, displayed improved fluorescence change in response to action potential trains and in addition showed robust responses to two-photon glutamate uncaging stimuli in dendritic spines. However, our studies also reveal significant limitations of GCaMP2 for monitoring neural activity in vivo.

## Results

### Responses of GCaMP-type GECIs to action potential trains

**Figure 2:**
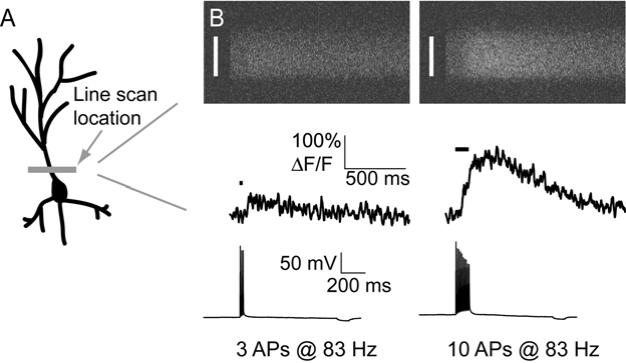
Recording backpropagating action potential responses from GECIs in hippocampal pyramidal cells. A, Schematic showing the linescan location at the base of the apical dendrite. B, Raw linescan images (top row) showing a dark period prior to shutter opening, followed by a shutter-open fluorescence baseline and action-potential (bottom row) evoked responses (left, 3 action potentials at 83 Hz; right, 10 action potentials at 83 Hz). Fluorescence time series (middle row) were obtained by averaging over the spatial extent of the dendrite (indicated by vertical white lines).

**Figure 3:**
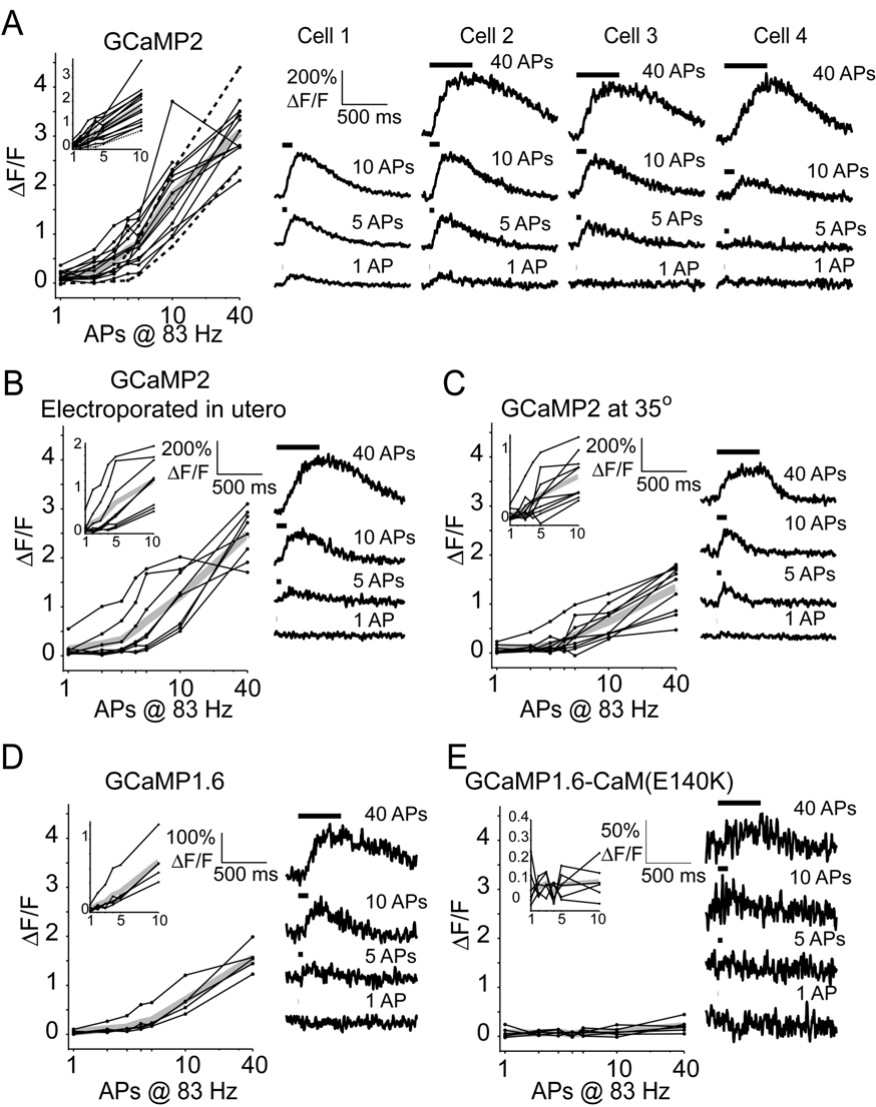
Action-potential evoked responses in GCaMP-based GECIs. A, Amplitudes of GCaMP2 responses for individual hippocampal pyramidal cells (thin lines, left) in response to trains of action potentials given at 83 Hz, and the mean across cells (thick gray line). Dashed lines show perforated-patch recordings. Inset shows same data for 1–10 action potentials on a linear x-axis. Example single-trial responses (right) from four cells to 1, 5, 10 and 40 action potentials at 83 Hz (indicated by horizontal black lines). B, Responses of individual cortical layer 2/3 pyramidal cells (thin lines) and the group mean (thick gray line) expressing GCaMP2 after *in utero* electroporation (see Materials and Methods). C, GCaMP2 responses from hippocampal pyramidal cells at 34.5–35.5°C. D,E, Responses of previous versions of GCaMP family GECIs. B–E, Same conventions as in A.

We made whole–cell recordings from GCaMP-expressing cultured hippocampal pyramidal neurons [31], [32] and in acute cortical brain slices at room temperature. Under baseline conditions GCaMP fluorescence was very low. For example, it was often difficult to image small dendritic branches and to detect dendritic spines. Action potentials were evoked by short current injections (3–5.5 nA, 2 ms). Our basic experiment comprised measuring GECI responses to high-frequency (83 Hz) action potential trains (Figure 2). Under our experimental conditions the peak Ca^2+^ accumulations are approximately proportional to action potential frequency [5], [32], [33]. We acquired linescans from the proximal apical dendrite (within 50 µm of the soma) (Figure 2). In cultured hippocampal neurons transfected with GCaMP2 and the cytoplasmic red protein mCherry [34] single action potentials caused clear fluorescence increases in some, but not all, neurons (Figure 3A). The average response to single action potentials was small (17±10% [mean±SD] ΔF/F across n = 13 cells). A train of 40 actions potentials (APs) at 83 Hz gave a robust response of 302±50% ΔF/F (n = 12 cells), close to GCaMP2's dynamic range measured in cuvettes [24].

GCaMP2 responses in layer 2/3 pyramidal cells in acute cortical brain slices (postnatal day 14–21, see Materials and Methods) were similar (1 AP response, 13±17% ΔF/F, n = 8; 40 AP response, 248±51% ΔF/F, n = 8) (Figure 3B) to the responses measured in cultured neurons. The recorded cells had healthy input resistances and resting potentials (see Materials and Methods) and apparently normal morphology. Thus, even though GCaMP2 was expressed at high concentrations for up to 4 weeks, the similar ΔF/F responses suggest that endogenous calmodulin did not interfere with the function of the calmodulin-based GCaMP2. Furthermore, GCaMP2 did not appear to degrade the health of the transfected neurons.

We next measured GCaMP2 responses near physiological temperature (34.5–35.5°). Consistent with faster calcium extrusion [35] and a narrower action potential, GCaMP2 responses were smaller (1 AP response, 6±8% ΔF/F, n = 10; 40 AP response, 134±48% ΔF/F, n = 10) (Figure 3C). GCaMP2 responses were also much faster (Figure 3C; room temperature: rise T_1/2_: 95±15 ms; decay T_1/2_: 483±127 ms, n = 13 cells; near-physiological temperature: rise T_1/2_: 73±15 ms; decay T_1/2_: 134±39 ms, n = 10 cells; all measurements for the 10 AP stimulus). The decay time of the GCaMP2 fluorescence transient is ∼2 fold slower than the decay time of [Ca^2+^] accumulations [35]. These values are in general agreement with GCaMP2 response kinetics measured in cerebellar granule cells in vivo following electrical stimulation [36].

We also tested GCaMP1.6 [37]; (see also [32]) and GCaMP1.6-CaM(E140K) . The E140K mutation is located in a calcium binding site and has been shown to increase the brightness of the sensor and decrease the affinity of the sensor for calcium [37]. GCaMP1.6 (Figure 3D) gave much smaller response amplitudes than GCaMP2 (1 AP, 4±4% ΔF/F, n = 5; 40 AP, 155±28% ΔF/F, n = 5) at room temperature. Single action potentials did not elicit clear responses above the noise. GCaMP1.6-CaM (E140K) gave even smaller responses (Figure 3E; 40 AP at 83 Hz, 21±13% ΔF/F, n = 6).

**Figure 4:**
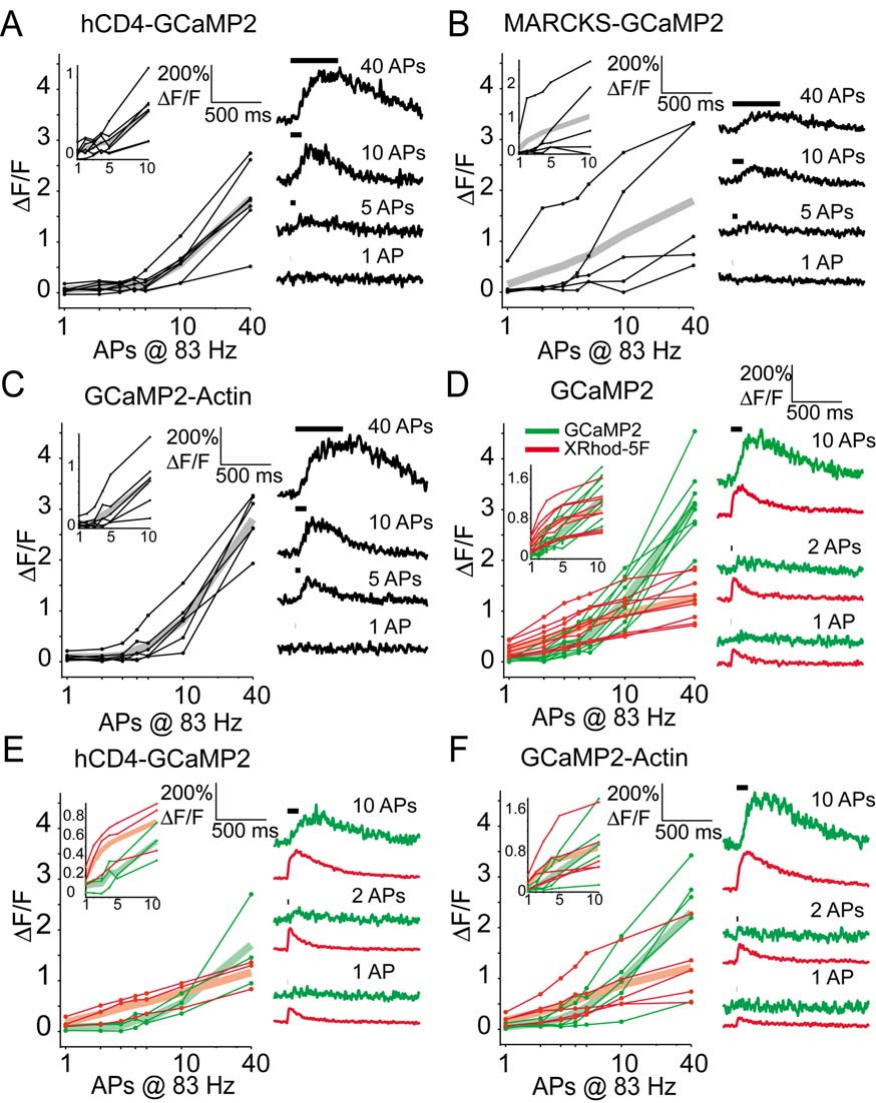
Action-potential evoked responses in GECIs targeted to subcellular locations. A, Amplitudes of the response to action potential trains at 83 Hz for the membrane-targeted GECI hCD4-GCaMP2 (left), for individual cells (thin black lines) and for the group mean (thick gray line). Insets show same data for 1–10 action potentials on a linear x-axis. Example traces (right) show single-trial responses to trains of action potentials at 83 Hz (indicated by horizontal black lines). B, Responses from the membrane-targeted construct MARCKS-GCaMP2. C, Actin-potential evoked responses from the GCaMP2-actin fusion. Conventions as in A. D–F, Action-potential responses measured simultaneously with GECIs (green curves, traces) and with the synthetic dye X-Rhod-5F (500 µM; red curves, traces). Cells were loaded with X-Rhod-5F for ≥20 min prior to data collection. Example traces show single-trial responses measured simultaneously from the green and red channels. Cells shown in D–F are different from those in Figure 3A and in panels A–C.

To better relate GECI fluorescence to changes in [Ca^2+^], we performed additional experiments in which we simultaneously measured responses from the GECIs and from the medium-affinity synthetic red calcium dye X-Rhod-5F, loaded through the patch pipette (Figure 4D). GCaMP2 responses were slower than X-Rhod-5F responses (GCaMP2: rise T_1/2_,100±11 ms, decay T_1/2_, 458±141 ms; X-Rhod-5F: rise T_1/2_, 29±6 ms, decay T_1/2_, 254±57 for X-Rhod-5F, n = 9 cells; all measurements for the 10 AP stimulus), consistent with GCaMP2 responses being slower than the underlying calcium dynamics. GCaMP2 response kinetics were similar to those measured in the absence of X-Rhod-5F (rise T_1/2_: 95±15 ms; decay T_1/2_: 483±127 ms, n = 13 cells; same data as above).

### GECIs targeted to subcellular locations

During action potential trains in pyramidal cells, the dominant source of dendritic calcium ion influx is through voltage-gated calcium channels in the plasma membrane [3], implying that peak Ca^2+^ concentrations are higher close to the membrane. By targeting GECIs to the plasma membrane, it may be possible to increase GECI responses and thereby to improve their ability to detect action potentials. We made and tested membrane-targeted versions of GCaMP2 (hCD4-GCaMP2 and MARCKS-GCaMP2; see Figure 1B). However, neither the hCD4-domain (Figure 4A, E) nor the MARCKS-domain (Figure 4B) membrane-targeted versions of GCaMP2 yielded an improvement in action-potential detection compared to cytosolic GCaMP2. Similarly, a GCaMP2-chicken β actin fusion protein, which targeted GCaMP2 to dendritic spines [38](Figure 1B), also failed to yield improvements in the GCaMP2 signal in response to action potentials (Figure 4C, F).

To eliminate the possibility that our subcellularly targeted GCaMP2 fusion proteins significantly perturbed the calcium channel or calcium handling machinery of the cell we performed additional experiments in which we simultaneously measured responses from the GECIs and from X-Rhod-5F, loaded through the patch pipette. X-Rhod-5F responses were similar in the three cases, indicating that global calcium influx and handling were unaffected by the presence of membrane-bound GECIs (Figure 4D–F).

### Quantifying GECI-based action potential detection

**Figure 5:**
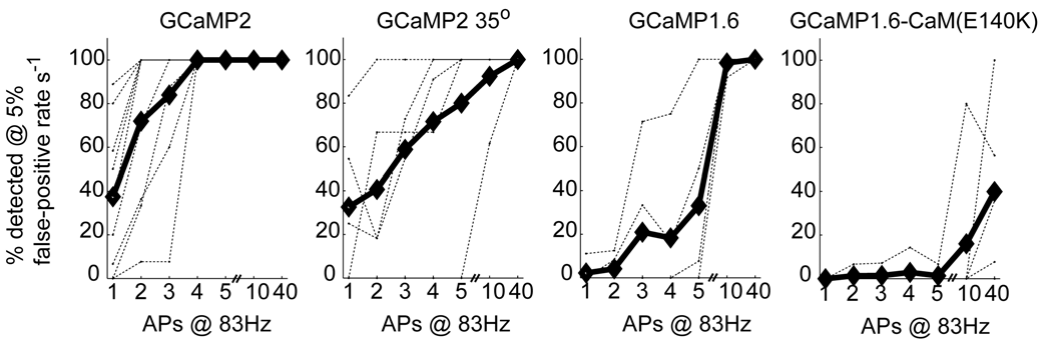
Accuracy of action potential detection. Results of simulations (see Materials and Methods) giving percentage of action potential trains (for the indicated numbers of action potentials at 83 Hz) that can be detected at a 5% false positive rate for 1 second of data (i.e., such that when a time series is divided up into subsequent intervals of 1 second length, 5% of these intervals will contain a false positive). Thin dotted lines show results for individual cells and the thick black line gives the group mean.

One promising area of application for GECIs is in all-optical monitoring of action potentials. It is therefore important to quantify the ability to infer action potentials from GECI fluorescence. We quantified our ability to detect action potentials under the highly-favorable conditions of brain slice recordings. A template-matching algorithm was able to detect single action potentials with nearly 100% certainty, provided the time of the action potential was known. However, when we simulated the case in which the time of the action potential was unknown, detection rates, given reasonable false-positive rates, dropped dramatically. For example, we determined the percentage of action potential trains (for trains of 1, 2, 3, 4, 5, 10 and 40 action potentials at 83 Hz) that could be detected at a 5% false positive rate during 1 second of data acquisition (i.e., such that when a time series is divided into subsequent intervals of 1 second length, 5% of these intervals will contain a false positive event). With trains of four or more action potentials, GCaMP2 at room temperature allowed us to detect 100% of the action potential trains (Figure 5). However, with a single action potential, GCaMP2 allowed detection of fewer than half of the action potentials. At near-physiological temperatures, it took about 5 action potentials at 83 Hz to achieve 80% detection rate with GCaMP2, and only the 40 action potential train gave 100% detection (Figure 5). Previous versions of GCaMP yielded lower levels of detection (GCaMP1.6, GCaMP1.6-CAM(E140K); Figure 5), illustrating the improvement of GCaMP2 over its predecessors.

### Spine NMDAR-mediated [Ca^2+^] transients detected by GCaMP2 targeted to the actin cytoskeleton

**Figure 6:**
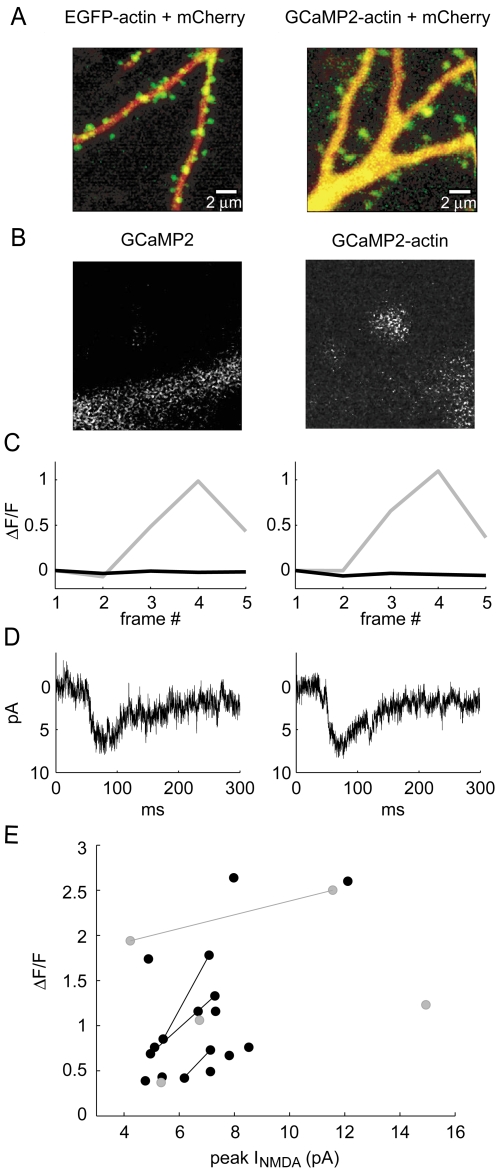
GCaMP2 and GCaMP2-actin uncaging responses in spines. A, Apical dendrite of a CA1 pyramidal cell (left) expressing EGFP-actin (green) and mCherry (red), showing an enrichment of EGFP-actin at spines (predominantly green) compared to dendrite (predominantly red). A GCaMP2-actin fusion (right) shows a similar spine enrichment in the apical dendrite of a different CA1 cell. B, Example images showing spine and dendrite fluorescence for GCaMP2 (left) and GCaMP2-actin (right). Each image shows the baseline frame prior to uncaging. Images are median filtered in a 3×3 pixel neighborhood. C, Traces show uncaging-evoked ΔF/F fluorescence signals for ROIs covering the spine (gray) and dendrite (black) for GCaMP2 (left) and GCaMP2-actin (right). Uncaging occurs at the start of the third frame. Traces correspond to the spines/dendrites shown in B. D, Traces showing NMDA-receptor currents in response to glutamate uncaging at the spines shown in B. Each trace shows an average of 6 trials. E, ΔF/F fluorescence change in spines versus peak NMDA-receptor current versus for GCaMP2 (gray circles) and GCaMP2-actin (black circles). Lines indicate measurements taken from the same spine at different uncaging powers.

NMDA-receptor mediated calcium accumulations can be imaged as a read-out of synaptic transmission [15], [16], [39]. We asked how well GCaMP2 fluorescence could signal NMDAR-mediated calcium transients in spines. We enriched GCaMP2 at spines by fusion to actin (Figure 6A). We then tested the performance of cytosolic GCaMP2 and GCaMP2-actin in response to two-photon uncaging of MNI-glutamate next to spines of pyramidal cells in hippocampal slice culture, in the presence of NBQX (to block AMPA receptors) and under low [Mg^2+^] conditions (see Materials and Methods). Cells were held in voltage-clamp at −70 mV and a single uncaging pulse of 0.2 ms was delivered after a short baseline imaging period (Figure 6C). Both GCaMP2 and GCaMP2-actin produced robust ΔF/F responses in response to NMDA receptor currents of ∼4–15 pA (GCaMP2 range: 37%–250% ΔF/F; GCaMP2-actin range: 39%–264% ΔF/F), with larger ΔF/F values corresponding to increased NMDAR current (Figure 6E). However, GCaMP2 and GCaMP2-actin were both quite dim and it was therefore sometimes difficult to locate spines (Figure 6B). Further, because GCaMP2-actin is targeted to the actin cytoskeleton and is not rapidly replaced by freely-diffusible cytosolic GECI, we found photobleaching to be a greater problem with GCaMP2-actin than with plain GCaMP2. In general, photobleaching is likely to present a greater problem with targeted GECIs than with their non-targeted counterparts, due to slowed fluorescence recovery after photobleaching.

### GECI brightness

**Figure 7:**
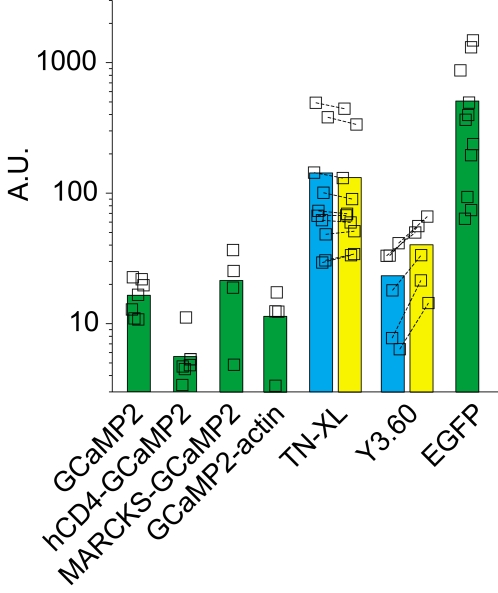
Brightness of the GECIs compared. GECIs were expressed (34–48 hours) in pyramidal cells and brightness was measured as the mean fluorescence intensity collected in linescan mode across the base of the apical dendrite. Each plot symbol shows a single cell, except for the FRET probes in which symbols connected by a dashed line indicate cyan and yellow channel measurements from the same cell. Illumination was 7 mW at the objective back aperture (see Materials and Methods for details).

An important property to consider in evaluating a GECI is its basal level of brightness. A GECI's signal-to-noise ratio, SNR ∼N^1/2^ ΔF/F, increases in proportion to the square root of the number (N) of photons collected and to the ΔF/F signal change. Maximizing the number of photons collected from the specimen is thus critical. What factors affect GECI brightness in a living neuron? The number of collected photons, N ∼ α c r, is proportional to GECI concentration (c) and to the photon rate (r), and to instrumentation properties (α). GECI concentration, c = k_f_/k_d_, is determined by the rate of functional protein formation (k_f_) and the rate constant of protein destruction (k_d_). At any moment in time the photon rate is proportional to quantum yield and decreases with the rate of quantum bleaching. Therefore, GECI brightness is affected both by properties of the cellular environment and by properties intrinsic to the GECI molecule. Here we made a rough relative measurement of brightness that confounds these different factors but that allows a gross comparisons across GECIs. We illuminated GECI-expressing pyramidal cells in hippocampal slice culture with 7 mW at the objective back aperture, and recorded the fluorescence intensity over a linescan image taken at the base of the apical dendrite (Figure 7; note that different filter sets were used for the FRET-based and the GFP-based GECIs; see Materials and Methods). GCaMP2 was about 30-fold dimmer than EGFP on average (it was for this reason that we co-transfected GCaMP2-related constructs with mCherry to easily identify transfected cells). The FRET-based TN-XL was the brightest GECI tested.

## Discussion

In neurons, transient changes in intracellular calcium accompany action potentials and are often used as a reporter of neuronal activity. Synthetic calcium dyes have been used both *in vitro* and in *vivo* [6], [7], [40], [41] in mammalian neurons to measure the calcium dynamics underlying neural activity, and have greatly improved our understanding of nervous system function. However, synthetic dyes suffer from several practical limitations, including: 1) they are difficult to load into populations of neurons in vivo; 2) they cannot readily be targeted to specific cell types or subcellular locations; and 3) they do not readily permit long-term chronic imaging *in vivo*. All these limitations can be potentially addressed by genetically-encoded calcium indicators (GECIs).

In this report we have quantitatively characterized the action potential responses of GCaMP-based GECIs under nearly ideal imaging conditions in the brain slice, and with the high expression levels possible with gene gun transfection and/or in utero electroporation. We also quantified the ability of GCaMP2 to allow detection of action potentials, under our particular expression and imaging conditions. At near-physiological temperatures, about 5 action potentials at 83 Hz were required to achieve an 80% detection rate (for a false positive rate of 5%) with GCaMP2. GCaMP2 did not allow consistent detection of single action potentials, even at room temperature where calcium extrusion kinetics are slower. It is therefore unlikely that GCaMP2 will allow faithful monitoring, at single action potential resolution, of a population of individual pyramidal neurons at mammalian physiological temperatures. At the moment, GCaMP2 may be suited to a preparation where neurons fire bursts of APs.

Cellular and subcellular targeting of GECIs holds great promise as a means to read out localized calcium signals. Many efforts have been made to target previously available GECIs to different subcellular locations, including to the plasma membrane [20], [25], [42]–[45]. None of the prior membrane-targeting studies, however, had the temporal resolution to monitor non-equilibrium differences between membrane-bound and cytosolic GECI signals, nor did any investigate GECI signaling of calcium influx mediated by action potential/voltage-gated calcium channel activity. To explore the effect of subcellular targeting of GECIs, we targeted GCaMP2 to various subcellular locations, including to the plasma membrane and to dendritic spines. Surprisingly, we found no improvement in signal change for all three different membrane-targeted GECIs.

Since the dominant source of action potential-induced dendritic Ca^2+^ influx is through voltage-gated calcium channels in the plasma membrane [3], it may be possible for GECIs targeted to the plasma membrane to sense higher peak [Ca^2+^]. In principle, therefore, by targeting GECIs to locations at or near the plasma membrane, it may be possible to achieve greater ΔF/F signals than with diffusible GECIs. Why, then, did membrane targeting not yield an improvement? One hypothesis is that these calmodulin-based sensors could interact with endogenous calmodulin [20], [25], [26], interfering with their function through unwanted binding reactions. It is also possible that the membrane-targeted GECIs did not show improvements due to effects of the targeting sequence(s) on folding and stability of the GECI, even though we used three different membrane targeting sequences. It is also possible that the slow kinetics of the GECI fluorescence response (∼100 ms) reflects calcium binding kinetics that are too slow to capture the [Ca^2+^] transient close to the membrane before dissipation by diffusion. Another possibility is that sensor proteins or their fluorophores are not stable in the local environment near the plasma membrane. Finally, fluorescent proteins targeted to the plasma membrane with different lipid anchors can show clustering into membrane microdomains [46] and it is not clear how our membrane-targeted GECIs were arranged with respect to the calcium channels. It remains an open question to what degree action potential detection can be improved by subcellular targeting of the GECIs.

Prior efforts to express GECIs in transgenic mice have yielded mixed results. Some transgenic mouse lines were found to have only very small GECI ΔF/F signals [20], [47] compared to *in vitro* measurements of the GECIs' dynamic ranges, consistent with earlier studies in invertebrates. This lead to speculations [26] that relatively high levels of GECI expression could overwhelm interference from endogenous calmodulin. In addition, it is possible that long-term expression of GECI calcium buffers could affect cell physiology or trigger silencing of GECI gene expression. However, we found robust GCaMP2 responses in juvenile mice that had been electroporated *in utero* at embryonic day 16. GCaMP2 was driven by the CAG promoter, which is known to give expression early in development [48]. Thus, expression of GCaMP2 in layer 2/3 pyramids for up to weeks during development did not prevent it from functioning properly and did not obviously interfere with the health of the expressing neurons. This result is consistent with studies finding calcium responses after long-term expression of camgaroo-2 and inverse pericam under the αCaMKII promoter [26], with a study showing functional GCaMP2 expression in cerebellum of transgenic mice under the Kv3.1 potassium channel promoter [23] and with a study showing long-term expression of functional GCaMP2 in cardiac myocytes [24].

Areas for improvement of GECIs include baseline brightness, dynamic range, and accelerating the response kinetics to Ca^2+^ to best match expected patterns of action potential activity. To further improve GCaMP2, it will be helpful to obtain the high-resolution structures of calcium-bound and calcium-unbound states, as well as to develop rapid screening and testing systems designed to assay responses to action potential-like stimuli. In particular, it will be valuable to have a screening system that allows measurement of an effective benchmark for quantifying action-potential responses (an “AP_50_”), as it is difficult to predict action potential responses from equilibrium measurements.

## Materials and Methods

### Molecular biology

The original GCaMP2 expression construct was obtained from M. Kotlikoff, and generated by J. Nakai[24]; TN-XL from O. Griesbeck (MPI, Germany)[21]; Yellow Cameleon Y3.60 from A. Miyawaki (RIKEN, Japan) [25]. All of the above constructs were driven by the CMV promoter in mammalian expression vectors. GCaMP2 was subcloned into the pCAGGS vector with the CAG promoter (CMV-enhancer, β-actin promoter, and regulatory element from the woodchuck hepatitis virus (WPRE) [48]). Subcellularly targeted constructs were driven by the CAG promoter. Three membrane-targeted versions of GCaMP2 were generated: (1) the first 41 amino acids of human MARCKS domain mutant (Met1-Val41) [49], which is myristoylated at Gly2 and double palmitoylated at Cys3Cys4, fused to the N-terminus of GCaMP2 (linker sequence AAAT); (2) a short MARCKS domain (Met1-Lys7) fused to the N-terminus of GCaMP2 (linker sequence AAAT); (3) the human hCD4 transmembrane protein (Met1-Arg421) fused to the N-terminus of GCaMP2 (linker sequence AAAT). To target GCaMP2 postsynaptically, chick actin was fused to the C-terminus of GCaMP2 [50] (linker sequence GGR). MCherry [34], which we used to co-transfect with GECIs, is in a pRK5 vector and a pCAGGS vector, for cultured hippocampal slices and *in utero* electroporation, respectively.

### Gene transfection and slice preparation

Hippocampal cultured slices were prepared from P7 rats as described [31]. Slices were transfected using gold particle-mediated biolistic gene transfer (Helios Gene Gun, BioRad) at 4–8 days in vitro. The amount of DNA used in each bullet preparation was between 1–20 µg per full length of tubing. All GECIs except FRET-based GECIs were co-transfected with mCherry to aid identification of GECI-expressing cells. Imaging experiments were performed 36–48 hours after the transfection. For the acute slice experiments shown in Figure 3B, GCaMP2 and mCherry DNA was introduced into mice by *in utero* electroporation as described [48]. Acute slices were prepared from positively transfected mice at P14-21. After isofluorane anesthesia and decapitation, acute coronal slices were cut in chilled solution containing (in mM) 110 choline chloride, 25 NaHCO_3_, 25 d-glucose, 11.6 sodium ascorbate, 3.1 sodium pyruvate, 2.5 KCl, 1.25 NaH_2_PO_4_, 0.5 CaCl_2_, and 7 MgCl_2_, saturated with 95% O_2_/5% CO_2_. Slices were then transferred to artificial cerebrospinal fluid (ACSF) containing (in mM) 127 NaCl, 2.5 KCl, 1.25 NaH_2_PO_4_, 25 d-glucose, 25 NaHCO_3_, 2 CaCl_2_, and 1 MgCl_2_ saturated with 95% O_2_/5% CO_2_, and were incubated at 34 degrees for ∼15 min before being cooled to room temperature. All experiments were conducted according to protocols approved by the Institutional Animal Care and Use Committee of Cold Spring Harbor Laboratory.

### Electrophysiology

We made recordings from both CA1 and CA3 cells in hippocampal slice culture, and cortical layer 2/3 pyramidal cells in acute brain slices. For measuring action-potential evoked GECI responses, we recorded from 62 CA1 pyramidal cells, 12 CA3 pyramidal cells, and 8 layer 2/3 cortical pyramidal cells (*in utero* electroporated, for GCaMP2 only) The brightness measurements reported in Figure 7 included another 45 CA1 pyramidal cells and 3 CA3 pyramidal cells.

Patch pipettes were pulled from borosilicate glass (standard wall with filament) and were 3–6 MΩ when filled with (in mM) 128 K-methylsulfate, 10 HEPES, 10 Na-phosphocreatine, 4 MgCl_2_, 4 Na_2_ATP, 0.4 Na_2_GTP, 3 ascorbic acid (pH 7.2, 293 mOsm). In experiments reported in Figure 4D–F, 500 µM X-Rhod-5F was added to the pipette solution. In some experiments reported in Figure 6, 30 µM Alexa-594 was added to the pipette solution for spine visualization. In perforated-patch experiments reported in Figure 3A, pipettes were tip-filled with patch solution containing ≤0.5 mg/mL amphotericin B (1% DMSO; Sigma). Liquid junction potentials were not corrected. Cells were accepted if they had resting potentials ≤−50 mV and input resistances of at least 100 MΩ for CA1 and layer 2/3 cells. CA3 cells had input resistances of 117±57 MΩ (mean±SD).

For recordings slices were transferred to an immersion-type recording chamber (after ≥1 hr incubation for acute slices) and perfused with ACSF comprising (in mM) 127 NaCl, 2.5 KCl, 1.25 NaH_2_PO_4_, 25 d-glucose, 25 NaHCO_3_, 4 CaCl_2_, and 4 MgCl_2_ saturated with 95% O_2_/5% CO_2_. For experiments with action-potential stimuli, the ACSF included 10 µM (R)-CPP (Tocris) and 10 µM NBQX (Sigma) to block glutamate receptors. For the uncaging experiments shown in Figure 6, the ACSF calcium and magnesium concentrations were changed to 2 mM and 0.1 mM, respectively, (R)-CPP was omitted, and tetrodotoxin (1 µM, Calbiochem), d-serine (10 µM, Sigma), and MNI-glutamate (2.5 mM, Tocris) were added to the bath.

For experiments without X-Rhod-5F, data collection began typically within 2–3 minutes of break-in. To prevent wash-out of GECI fluorescence, most experiments were terminated within 20–25 minutes of break-in. In experiments measuring both GECI and X-Rhod-5F responses, data collection began after a dye-loading period of 20–23 minutes and continued until ≤43 min after break-in. Action potentials were triggered by current injections (3–5.5 nA, 2 ms) through the patch pipette. Trials were repeated at 0.1 Hz. Unless indicated otherwise, experiments were performed at room temperature (21–24°).

### Imaging and uncaging

We imaged on a custom-built two-photon microscope using ScanImage software [51] and an Olympus 60×, 0.9 NA LUMPlanFI/IR objective. For imaging and glutamate uncaging we used two Ti:sapphire lasers (Mira, Coherent, Santa Clara, CA; and MaiTai, Spectra Physics, Mountain View, CA). For imaging we tuned one laser to 910 nm, 960 nm or 810 nm (as indicated). For glutamate uncaging we used a wavelength of 720 nm. Fluorescence was collected in two channels in both epi- and transfluorescence mode [52] using four photomultiplier tubes (Hamamatsu, Hamamatsu City, Japan). For GCaMP2-based GECIs and EGFP, we separated fluorescence into “green” and “red” channels with 565 nm dichroics and BG22 (green channel) and HQ620/90 (red channel) emission filters. For the FRET-based GECIs (Figure 7) we separated fluorescence with 505 nm dichroics and HQ480/80 (“cyan” channel) and HQ535/50 (“yellow” channel) emission filters. For most experiments, images were acquired by scanning in linescan mode (500 Hz) across a location at the base of the apical dendrite (Figure 2A). Fluorescence time series were then obtained by averaging across the spatial extent of the dendrite along the line (Figure 2B). For glutamate uncaging experiments shown in Figure 6, images were acquired in framescan mode (256×256, 2 ms/line). The uncaging stimulus was 720 nm illumination for 0.2 ms at 100–135 mW.

We report time series as ΔF/F = [(F-F_D_)-(F_0_-F_D_)]/(F_0_-F_D_), where F is the raw fluorescence signal, F_D_ is the mean PMT “dark signal” recorded with the laser shutter closed, and F_0_ is the mean fluorescence signal in a baseline period prior to the action potential stimuli.

The response amplitude on a given trial was measured as the mean of a 30 ms window of the ΔF/F time series, which was centered on the peak of the smoothed (50-ms moving average) mean response for that cell and stimulus condition. Rise T_1/2_ was measured as the time between the onset of current injection and the half-maximal response. Decay T_1/2_ was measured as the time between the peak response and the decay back to half-maximum response. For display, example traces were filtered with a Savitzky-Golay filter of order 2 and span 30 ms. All analysis was performed with MATLAB (Mathworks, Natick, MA).

For the brightness measurements reported in Figure 7, each cell was first patched to confirm that it met our resting potential and input resistance criteria. The pipette was pulled off from the cell in <1 min after break-in, and the brightness was then measured. Illumination was 7 mW at the objective back aperture, at 810 nm for the FRET probes and at 910 nm for EGFP and the GCaMP2 probes. Filter sets were the same as those described earlier.

### Action potential detection

We quantified our ability to detect the presence of action potential responses for each of several GECIs, using a template matching method. The mean response for each cell at each action potential stimulus (i.e., 1, 2, 3, 4, 5, 10 and 40 action potentials at 83 Hz) was used as a template. For each sweep, we computed the cross-covariance sequence of the sweep with the corresponding template (using the first 1.6 s of each). We then recorded the peak of the cross-covariance sequence over an interval of 1 s (lags of −98 ms to +900 ms). We compared these cross-covariance peaks to those obtained by cross-correlation of the template with mock baseline data. Mock baseline data for each cell were obtained by concatenating short segments of actual baseline fluorescence data from the period prior to action potentials. The order of the baseline segments was randomly permuted in order to make 1000 sets of baseline data. We took the peak of the cross-covariance sequence for each mock baseline sweep and histogrammed all 1000. For each cell and stimulus, we defined a criterion cross-covariance peak value to be the 95^th^ percentile of the mock baseline cross-covariance peaks. Detection accuracy was then defined as the percentage of real sweeps with cross-covariance peaks exceeding the criterion. Each real sweep, mock baseline sweep, and the template were smoothed with a 10-ms moving average.
